# The epigenetic-immune-microbiome axis in early life: reprogramming the origins and endotypes of pediatric asthma

**DOI:** 10.3389/fimmu.2026.1835821

**Published:** 2026-08-03

**Authors:** Fu Zhang

**Affiliations:** Department of Pediatrics, Guangyuan Central Hospital, Guangyuan, Sichuan, China

**Keywords:** epigenetics, immune endotypes, immunometabolism, maternal immune activation, microbiome-gut-lung axis, pediatric asthma, trained immunity

## Abstract

Pediatric asthma is a highly heterogeneous inflammatory syndrome characterized by distinct immune endotypes. The inception of asthma predominantly occurs during the first 1,000 days of life, a critical window of immunological plasticity. Within this critical window, the developing immune system is continuously shaped by dynamic interactions between environmental exposures, the microbiome, and host genetics. Recent advances highlight the epigenetic-immune-microbiome axis as the central mechanism orchestrating these interactions. This review provides a comprehensive synthesis of how prenatal maternal imprinting (including maternal immune activation, nutrition, and psychological stress) and postnatal “second hits” (such as respiratory viral infections and gut-lung microbial dysbiosis)trigger the epigenetic reprogramming of airway epithelial cells and innate/adaptive immune cells. We specifically emphasize the paradigm of “trained immunity” in macrophages and dendritic cells, alongside the hyperactivation of group 2 innate lymphoid cells (ILC2s), as fundamental drivers of asthma pathogenesis. In parallel, we outline the epigenetic and metabolic networks (immunometabolism) that govern T-cell polarization (Th1/Th2/Th17/regulatory T cells) and airway mucosal barrier dysfunction. Finally, we evaluate the translational potential of these multi-omics signatures, discussing how non-invasive approaches like nasal epigenomic profiling may eventually aid in stratifying clinical endotypes and inform the development of future mechanism-based therapeutics.

## Graphical Abstract

**Figure:**
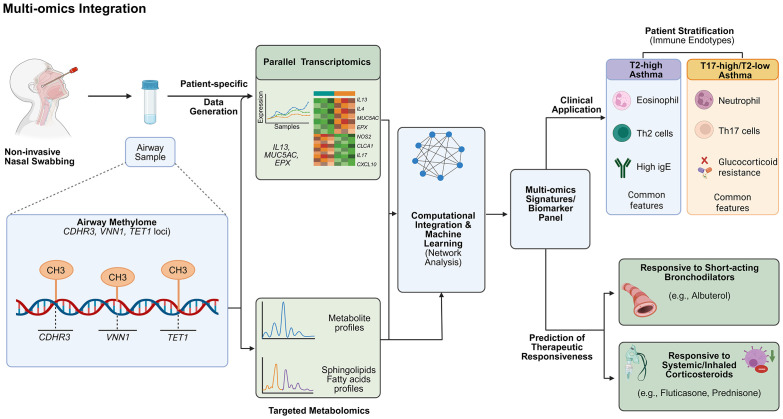
Figure 3 is designated as the Graphical Abstract. It illustrates the translational workflow from non-invasive nasal swabbing to multi-omics data generation (parallel transcriptomics, targeted metabolomics, and airway methylome profiling), culminating in AI-driven integration and machine learning. This computational pipeline shows potential for facilitating the stratification of immune endotypes (T2-high vs. T17-high/T2-low) and assisting in the prediction of therapeutic responsiveness (e.g., Albuterol, Corticosteroids) for pediatric asthma precision medicine.

## 1 Introduction: paradigm shifts in pediatric asthma origins

### 1.1 Clinical heterogeneity and early-life inception of asthma

Asthma represents the most prevalent chronic pediatric respiratory disease globally, posing a substantial clinical and economic burden on healthcare systems (1, 2). Traditionally viewed as a singular, immunoglobulin E (IgE)-mediated allergic condition characterized by coughing, wheezing, and reversible airflow obstruction, asthma is now widely recognized as a complex, heterogeneous syndrome (3). It comprises multiple clinical phenotypes driven by distinct pathophysiological endotypes. These range from the classical T helper 2 (Th2)-high, eosinophilic, and highly atopic asthma to Th17-driven, non-atopic, or obesity-associated asthma phenotypes (4–7). The developmental trajectory of pediatric asthma typically begins in early childhood, often manifesting initially as recurrent preschool wheezing, a highly heterogeneous condition itself that frequently foreshadows lifelong respiratory morbidity (8–11).

Epidemiological and mechanistic studies increasingly converge on the “developmental origins of health and disease” (DOHaD) hypothesis. This framework posits that the inception of asthma is deeply rooted in the *in utero* and early postnatal environments (12–16). Compelling evidence from large birth cohorts indicates that low birth weight and impaired fetal growth trajectories directly correlate with aberrant lung function and increased respiratory symptoms in adulthood, pointing toward intrauterine adaptive mechanisms (13, 17). During these critical developmental windows, often referred to as the first 1,000 days of life, the infant’s respiratory and immune systems are functionally immature and highly vulnerable to environmental insults (18–21). These early-life exposures can permanently alter lung growth trajectories, airway dysanapsis, alveolarization, and immune maturation, thereby dictating long-term respiratory health and determining disease susceptibility across the entire lifespan (11, 22–24).

### 1.2 Epigenetics as the “memory” bridge between environment and immunity

While genetic predisposition (e.g., polymorphisms in the 17q21 locus, *GSDMB*, *ORMDL3*) plays an undeniable role in asthma susceptibility (9, 25–28), twin studies powerfully demonstrate that while total IgE levels and general atopic tendency are heavily genetically determined, the specific offending allergens and severity manifestations are heavily skewed by environmental and epigenetic factors (28). The rapid global increase in asthma prevalence over the past few decades cannot be explained by genomic alterations alone (29–31). Epigenetics (defined as heritable yet reversible modifications that regulate gene expression without altering the underlying DNA sequence) has emerged as the crucial mechanistic bridge linking environmental exposures to long-term immune dysregulation (5, 15, 24, 32–34).

Epigenetic mechanisms, predominantly DNA methylation (DNAm) at cytosine-phosphate-guanine (CpG) dinucleotides, histone modifications (e.g., acetylation by histone acetyltransferases [HATs] or methylation by histone methyltransferases), and non-coding RNAs (ncRNAs), are highly responsive to early-life stimuli (24, 32, 35–37). Environmental epigenetics highlights how tobacco smoke, microbial allergens, and dietary methyl donors orchestrate aberrant DNA methylation and chromatin alterations (29). These modifications drive a complex early-life reprogramming of adaptive immune T-cell responses, innate dendritic cell (DC) function, macrophage activation, and non-immunologic mechanisms like airway wall remodeling (29, 31, 38–40). Crucially, these epigenetic “scars” can persist for years or even decades. They act as a durable molecular memory that determines the host’s threshold for immune tolerance versus hyperinflammation upon subsequent encounters with allergens, viruses, or atmospheric pollutants (5, 40–42).

### 1.3 Scope of the review

This review aims to meticulously decipher the intricate web of the epigenetic-immune-microbiome axis in the pathogenesis of pediatric asthma. We propose an innovative logical model transitioning from prenatal maternal and paternal imprinting (including stress, nutrition, and systemic inflammation) to postnatal microbial and viral exposures, culminating in profound mucosal immunity dysfunction. By integrating recent breakthroughs in trained immunity, immunometabolism, and multi-omics integration, we seek to elucidate the exact molecular mechanisms driving the evolution of distinct asthma endotypes. Ultimately, we outline how these immunological insights provide a robust framework for precision medicine, facilitating early risk stratification and the development of targeted immunomodulatory interventions.

## 2 *In utero* programming: maternal-fetal immune crosstalk and epigenetic imprinting

### 2.1 Epigenetic imprinting of maternal and paternal milieu (nutrition, obesity, and stress)

**Figure 1:**
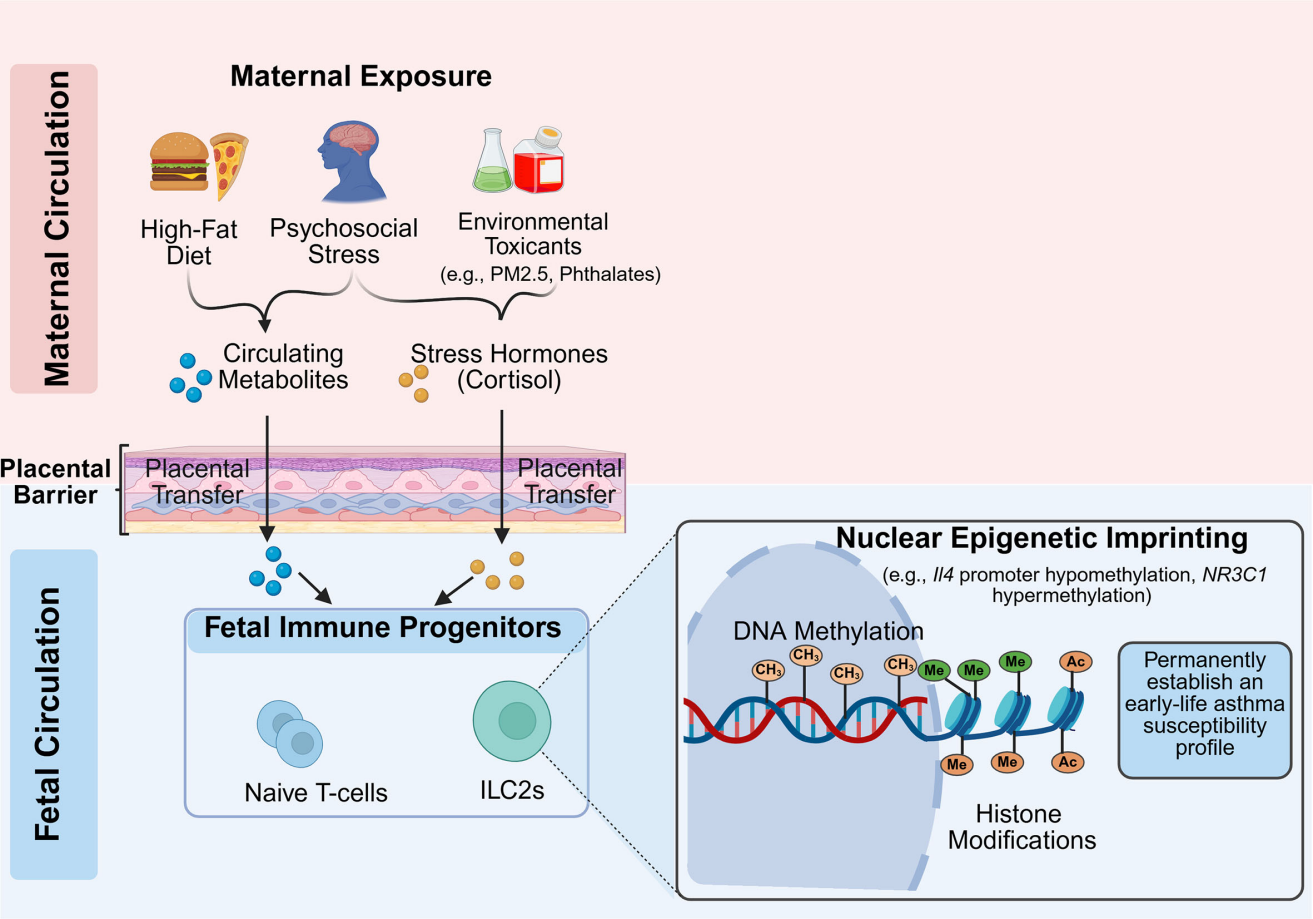
The *in utero* epigenetic imprinting. Diagram illustrating the maternal-fetal interface across the placental barrier. The schematic demonstrates how maternal exposures (e.g., high-fat diets, psychosocial stress, and environmental toxicants) generate circulating metabolites and stress hormones (like cortisol) that cross the placental barrier into the fetal circulation. Upon entering fetal immune progenitors (e.g., naive T cells and ILC2s), these factors act directly within the nucleus. The magnified inset details the DNA double helix, specifically highlighting the physical addition of DNA methylation groups and histone modifications that permanently establish an early-life asthma susceptibility profile.

The maternal environment during pregnancy exerts profound intergenerational, and potentially transgenerational, effects on the fetal immune system (14, 35, 43, 44) (**Figure 1**). Nutritional imbalances, such as maternal obesity, high-fat diets, micronutrient deficiencies, or altered folate status, can epigenetically skew fetal immune trajectories (45–47). For instance, elegant murine models demonstrate that maternal high-fat diet consumption profoundly exacerbates allergic asthma in adult offspring (46). Mechanistically, in these murine models, this hyperresponsiveness is driven by DNA hypomethylation in the *IL4* promoter region of naive CD4+ T cells, which dramatically lowers the activation threshold for a Th2-biased immune response upon later allergen challenge (46). Additionally, genome-wide studies indicate that DNAm at birth links to specific total IgE trajectories from birth to adolescence, highlighting ethnicity-specific methylation quantitative trait loci (methQTL) such as cg16711274 mapped to the *MINAR1* gene (27). In addition, seasonal variations during gestation (e.g., latitude-dependent winter births) permanently alter DNAm at differentially methylated regions (DMRs) corresponding to genes involved in airway inflammation like *LTB4R* (48).

Extensive reviews of nutrition in the first 1,000 days reveal that while unhealthy maternal dietary patterns disrupt fetal lung development, adherence to a Mediterranean diet during pregnancy has been robustly associated with a lower risk of allergic sensitization, wheezing, and asthma in offspring up to two years of age (44). Similarly, breastfeeding dynamically shapes the early respiratory and gut microbiomes, exerting beneficial effects against respiratory infections, although its absolute protective effect against atopic asthma remains debated (44). Additionally, emerging natural therapies such as the ketogenic diet have shown promise in preclinical models of obesity-associated asthma by directly reducing airway inflammation, modulating intestinal flora, and modifying epigenetic markers, presenting a novel dietary intervention strategy for this specific endotype (45). Evidence also suggests that folate deficiency is significantly associated with lung function impairment in asthmatic children, manifested by lower forced vital capacity (FVC) and forced expiratory volume in 1 second (FEV1), pointing to the critical role of methyl donors in epigenetic maintenance (47). Conversely, *in utero* dietary interventions can confer protection; maternal vitamin C supplementation given to pregnant smokers has been shown to alter buccal DNA methylation at specific disease-associated CpGs (e.g., *SLC25A37*) in 5-year-old children, correlating directly with significantly improved forced expiratory flow (FEF25-75) (11, 49).

Maternal psychological stress and exposure to violence also significantly influence fetal immune programming. Stress triggers the hypothalamic-pituitary-adrenal (HPA) axis, elevating systemic cortisol levels that cross the placental barrier and induce epigenetic modifications in the fetus (7, 50, 51). Prenatal stress has been mechanistically linked to the hypermethylation of the *NR3C1* gene (encoding the glucocorticoid receptor) in the hippocampus. This methylation decreases glucocorticoid receptor expression, leading to decreased negative regulation of the HPA axis, dysregulated cortisol production, and a subsequent skewing toward Th2 cytokine dominance in the offspring, thereby contributing to increased asthma susceptibility (51).

Interestingly, recent data from the RHINESSA cohort reveals that paternal exposures also critically impact offspring. An epigenome-wide association study (EWAS) demonstrated that a father’s adolescent body silhouette (especially during the pubertal “voice break” window) is strongly associated with offspring asthma and lung function through differential DNA methylation at specific loci, including *KCNJ10*, *FERMT1*, and imprinted genes like *VTRNA2-1* (52). Moreover, specific genetic polymorphisms such as those in the *ADAM33* gene exhibit profound paternal imprinting. The paternally transmitted GGGCTTTCGCA haplotype in *ADAM33* is significantly associated with severe airway hyperresponsiveness to methacholine in asthmatic children, suggesting a highly targeted epigenetic paternal transfer of respiratory risk (42). To directly study this early programming, researchers have successfully utilized the placental amniotic epithelial methylome as a highly conserved, non-invasive proxy to investigate fetal airway vulnerability to maternal smoking and asthma (53).

### 2.2 Maternal immune activation and transgenerational immune reprogramming

Maternal immune activation (MIA) secondary to acute viral infections, chronic asthma, or systemic hyperinflammation during pregnancy critically impacts fetal immune ontogeny (54–57). Asthmatic mothers provide a unique inflammatory intrauterine environment that directly alters fetal lung immunity, an effect independent of shared genetic loci (58). Preclinical studies demonstrate that in murine models, maternal asthma imprints fetal lung group 2 innate lymphoid cells (ILC2s) via enhanced glucocorticoid signaling (54). The offspring of asthmatic mothers exhibited persistently altered chromatin accessibility, particularly at glucocorticoid receptor-binding regions within lung ILC2s, detectable both at the fetal stage and well into adulthood. This prenatal epigenetic remodeling leads to ILC2 hyperactivation, increased local IL-5 and IL-13 production, and vastly worsened allergic airway inflammation in adulthood (54, 55). Furthermore, pre-conception parental infections exert long-lasting impacts; a history of paternal childhood tuberculosis has been epidemiologically associated with a substantially higher asthma risk in future offspring, suggesting that infection-induced epigenetic reprogramming of immune defenses may transfer across generations (59). Similar profound immune dysregulation driven by epigenetic modifications (DNA methylation and microRNA alterations) has been observed in neonates vertically infected with human immunodeficiency virus (HIV), skewing T-cell differentiation and exhausting antiretroviral therapy responses (60).

Fetomaternal immune crosstalk during helminth or viral infections massively modifies neonatal T-cell priming and DC phenotypes. Exposure to maternal schistosomiasis, even without direct vertical transmission of the parasite, skews offspring CD4+ T-cell responses towards an IL-4/B-cell-dominant phenotype and alters CD8+ T-cell responses through sustained, epigenetically encoded modifications to DC functions (61). Similarly, severe maternal SARS-CoV-2 infection (COVID-19) is associated with elevated maternal pro-inflammatory cytokines (IL-6, IL-17A) and induces widespread DNA methylation changes (over 3000 differentially methylated sites) in infant buccal cells. These robust epigenetic alterations severely impact genes relevant to synaptic and immune pathways, correlating strongly with neurodevelopmental and respiratory delays at 12 months of age (57, 62–65). In addition, diabetic pregnancies combined with COVID-19 compound this effect. Trans-generational programming during gestation via pro-inflammation, hypoxia, and subsequent epigenetic modifications based on a disrupted intra-uterine milieu is proposed to “program” lifelong susceptibility in the offspring (64). These findings suggest that maternal immunity strong influences the baseline inflammatory set-point of the neonate.

### 2.3 Environmental toxicants and epigenetic inheritance

Prenatal exposure to environmental pollutants, such as fine particulate matter (PM2.5), black carbon, and endocrine-disrupting chemicals (EDCs) like phthalates, significantly escalates the risk of childhood asthma and airway hyperresponsiveness (16, 19, 21, 66). Endocrine disruptors can act as faulty hormonal imprinters during the critical perinatal window, altering cytokine production and immune cell ratios by binding to developing hormone receptors on lymphocytes and mast cells (35). Strikingly, maternal exposure to butyl benzyl phthalate (BBP) promotes allergic airway inflammation over two subsequent generations (67). This robust transgenerational effect is mediated by BBP-induced global DNA hypermethylation in CD4+ T cells, which specifically represses the transcription of the GATA-3 repressor zinc finger protein 1 (*Zfpm1*). In murine models, the silencing of *Zfpm1* enhances Th2 differentiation and exacerbates airway inflammation (67). Additionally, prenatal exposure to di-(2-ethylhexyl) phthalate (DEHP) causes global placental DNA hypomethylation, linking early respiratory issues with broader systemic and neurological anomalies (68).

Airborne particles also exert potent, rapid epigenetic effects. Indoor environments, prominently characterized by secondhand smoke and specific allergens, heavily influence asthma morbidity (69). Conversely, outdoor ambient air pollution triggers oxidative stress pathways and allergic sensitization (21, 70). In pediatric populations, the presence of specific genetic polymorphisms related to antioxidant enzymes significantly heightens susceptibility to pollution (22, 30, 66). Personal exposure to black carbon induces rapid DNA demethylation of key pro-inflammatory genes (e.g., *IL4* at CpG-48, *NOS2A* at CpG+5099), amplifying asthmatic responses. This effect is particularly pronounced in genetically susceptible or already seroatopic children sensitized to indoor allergens like cockroach antigens (36, 71). Interestingly, while physical activity generally improves lung function, active urban children exposed to high black carbon levels display specific demethylation in the *FOXP3* promoter, suggesting that air pollution forces compensatory epigenetic alterations in Treg functionality to suppress airway inflammation (72). Moreover, long-term exposure to PM2.5 positively correlates with hypermethylation of the *IFNG* (IFN-γ) promoter region in CD4+ T cells in pediatric allergic rhinitis, directly suppressing protective Th1 responses (73).

Beyond airborne particulates, cumulative exposure to heavy metals like cadmium (Cd) following early-life respiratory infections has been shown to potentiate profound metabolic reprogramming. Preclinical models illustrate that low-dose Cd exposure following infantile respiratory syncytial virus (RSV) infection significantly disrupts the histamine, histidine, and urea cycle pathways, while concurrently activating the mechanistic target of rapamycin complex-1 (mTORC1). This metabolomic disruption substantially exacerbates long-term fibrotic and inflammatory lung damage (74). Continuous early-life inhalation exposure to mine tailings dust similarly triggers profound epigenetic alterations that promote epithelial-to-mesenchymal transition (EMT) and intense eosinophilic inflammation in the developing lung (75).

## 3 The postnatal “second hit”: mucosal immunity, viral infections, and the microbiome

### 3.1 Viral-induced immune reprogramming and airway remodeling

Early-life lower respiratory tract infections (LRTI), particularly those caused by RSV, human rhinovirus (HRV), and human metapneumovirus (HMPV), are universally recognized as the major postnatal risk factors for the inception of asthma (76–81). The critical linkage between early-life viral wheezing and subsequent asthma risk was initially established by landmark inception cohorts, including the Tucson Children’s Respiratory Study (TCRS), COAST, and the Urban Environment and Childhood Asthma (URECA) study (82–86). While these foundational cohorts were not primarily designed as multi-omic epigenetic investigations, they delineated the precise developmental exposure windows, linking early microbial and viral trajectories to epithelial barrier function and immune maturation. These windows now perfectly underpin our current mechanistic models of asthma inception (87–90). A fundamental driver of this vulnerability is the state of neonatal immunological maturity. The rapid development of the immune system immediately after birth creates an environment that favors immunosuppression over robust antiviral innate responses, leading to poor viral clearance (79). Neonates exhibit relative impairments in Th1-polarizing cytokine production and deficient expression of critical antimicrobial proteins (APPs) such as lactoferrin and surfactant proteins (SP-A, SP-D), which normally enhance pathogen phagocytosis by alveolar macrophages (91, 92).

**Figure 2:**
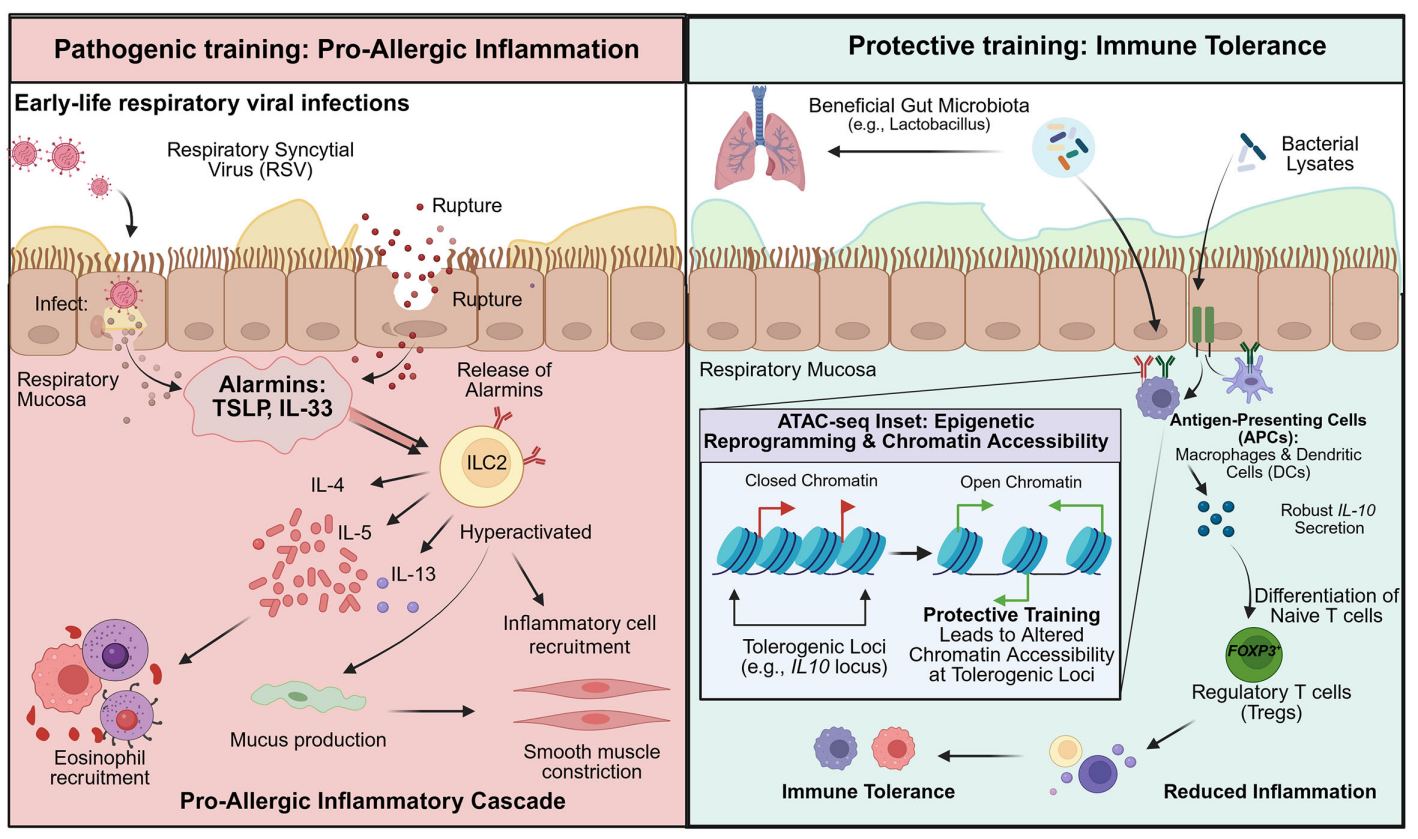
Postnatal “second hits” and the dichotomy of trained immunity. A contrasting schematic illustrating divergent innate immune memory pathways in the respiratory mucosa. (Left Panel) Pathogenic training: Early-life respiratory viral infections (e.g., RSV) cause epithelial cell rupture and the massive release of alarmins (TSLP, IL-33), which directly hyperactivate ILC2s and drive a pro-allergic inflammatory cascade. (Right Panel) Protective training: Exposure to beneficial gut microbiota (e.g., *Lactobacillus*) or bacterial lysates acts on antigen-presenting cells like macrophages and dendritic cells. The magnified inset provides a graphical representation of ATAC-seq concepts, showing altered chromatin accessibility (open chromatin states) at tolerogenic loci. This epigenetic reprogramming leads to the robust secretion of IL-10, subsequently inducing regulatory T cells (Tregs) and steering the system towards immune tolerance.

The neonatal immune system is intrinsically skewed towards a tolerogenic, Th2/Th17-biased phenotype (92–95). For example, during neonatal HMPV infection, neonatal Th1 function is actively restrained by surface PD-1 expression, and Tregs provide extrinsic regulation via TGF-β production that heavily limits Th1 formation compared to adults (93). However, this relative functional immaturity results in aberrant and potentially pathogenic responses to subsequent viral insults. RSV infection directly damages the vulnerable airway epithelium, disrupting tight junction integrity and triggering the massive release of epithelial-derived “alarmins,” including thymic stromal lymphopoietin (TSLP), IL-33, IL-25, and high mobility group box 1 (HMGB1) (41, 96, 97). These alarmins potently and directly activate ILC2s, which serve as the dominant innate counterparts to adaptive Th2 cells. Activated ILC2s rapidly secrete copious amounts of IL-4, IL-5, and IL-13, driving immediate mucus metaplasia, severe eosinophilia, and airway hyperresponsiveness completely independently of adaptive Th2 cell engagement (78, 97) (**Figure 2**).

Notably, early-life HRV or RSV infections leave an enduring epigenetic “scar” on respiratory epithelial and local immune cells. Studies utilizing primary nasal epithelial cells from asthmatic children infected with Rhinovirus-16 (RV-16) identified 471 CpGs with HRVI-induced asthma-specific modified DNA methylation, severely impacting host immune response genes such as *BAT3* and *NEU1* (98). This leads to an exaggerated “asthma-like” immune phenotype upon subsequent heterologous viral infections or environmental allergen exposures (78, 98–101). For instance, sequential infection with RV-A1B at day 6 of life followed by heterologous RV-A2 at day 13 induces an intensified, ILC2-dependent asthma-like phenotype, massive mucus metaplasia (elevated *Muc5ac* expression), and airway hyperresponsiveness (78). In human cohorts, reduced DNA methylation at the *PRF1* (perforin) enhancer in children is significantly associated with a history of severe RSV bronchiolitis in infancy, underscoring a lasting epigenetic defect in cytotoxic immunity (80).

Beyond respiratory viruses, exposure to specific viruses like Hepatitis C Virus (HCV) and Epstein-Barr Virus (EBV) fundamentally reprograms immune developmental trajectories. HCV exposure in neonates heavily alters adaptive B cells (triggering early B cell maturation, IgA plasma cell shifts, and CD40 upregulation) and skews innate myeloid and NK cell profiles (102). Latent EBV seropositivity has been causally linked to profound immune dysregulation and drastically increased mortality in acute pediatric sepsis, mediated by hyperferritinemia and macrophage activation syndrome (103).

The host’s genetic architecture intimately interacts with these viral exposures. For instance, specific polymorphisms in the human rhinovirus C (RV-C) receptor, cadherin-related family member 3 (*CDHR3*), significantly dictate receptor epithelial expression levels and activation, strictly correlating with asthma development and exacerbation severity in children (8). In the context of recent global pandemics, the interplay between viral infection and pediatric immunity has been further highlighted by SARS-CoV-2. While children generally exhibit milder COVID-19 symptoms, partially attributed to trained immunity from regular childhood vaccinations and differential innate cytokine responses (65, 104), a subset develops Multisystem Inflammatory Syndrome in Children (MIS-C). MIS-C represents a severe, hyperinflammatory, and immunosuppressed condition characterized by an over-exaggerated innate immune response, selective cytokine storm, and T-cell suppression (63). Recent multi-omics analyses reveal that MIS-C is associated with massive tissue injury, quantifiable via cell-free DNA (cfDNA) derived from non-hematopoietic tissues and innate immune cells, offering a highly sensitive biomarker for mapping acute, virus-induced multiorgan inflammatory damage (105).

### 3.2 Gut-lung axis: microbial colonization and immune tolerance

The sequential colonization of the infant microbiome during the first critical years of life plays an indispensable role in educating and calibrating the developing immune system (20, 106–109). Dysbiosis, often precipitated by cesarean delivery, exclusive formula feeding, or early-life perinatal broad-spectrum antibiotics, severely disrupts this vital educational process, significantly increasing asthma and food allergy risks (44, 110, 111). The foundational evidence for the gut-lung axis in pediatric asthma emerged from landmark longitudinal birth cohorts. For instance, the Canadian Healthy Infant Longitudinal Development (CHILD) cohort conclusively demonstrated that infants at increased risk of asthma exhibit a reduced abundance of specific bacterial taxa (e.g., Faecalibacterium, Lachnospira) within the first 100 days of life (112). Subsequent investigations from the Copenhagen Prospective Studies on Asthma in Childhood (COPSAC), Vitamin D Antenatal Asthma Reduction Trial (VDAART), and Protection against Allergy: Study in Rural Environments (PASTURE) cohorts corroborated these findings, establishing that aberrant gut microbiome maturation trajectories during infancy are profoundly associated with later asthma development (113–115). Notably, the PASTURE farm cohort further demonstrated that this maturation pattern may partly explain the protective ‘farm effect’ (115). Seminal studies show that infants who later develop food allergies harbor lower abundances of *Bifidobacterium* and *Clostridia* species. The early bacterial microbiome directly protects against food allergies through the production of anti-inflammatory metabolites that induce Tregs and impart trained immunity (110). In newborn macaques, early-life broad-spectrum antibiotic exposure induces massive neutrophil senescence, macrophage dysfunction, and renders the lungs highly susceptible to fatal bacterial pneumonia, a dysbiosis completely reversible via fecal microbiota transfer (110). Moreover, age-sensitive contact with commensal microbes is vital; germ-free mice accumulate highly pathogenic invariant natural killer T (iNKT) cells in the lungs due to elevated *CXCL16* expression, heavily exacerbating allergic asthma (108).

The emerging concept of the “Gut-Lung Axis” highlights how intestinal microbiota residing distally in the gastrointestinal tract can profoundly modulate pulmonary immunity through the systemic distribution of microbially-derived metabolites, such as short-chain fatty acids (SCFAs), tryptophan derivatives, and inosine (106, 116). For example, microbiota-derived inosine has been shown to promote protective CD8+ T-cell responses against respiratory viruses by epigenetically regulating *TCF1* expression via the crucial transcription factor NFIL3 (106). In mice, early-life antibiotic exposure reduces intestinal inosine, leading to intrinsic defects in CD8+ T-cell proliferation and impaired tissue-resident memory formation, a defect rescuable by *Bifidobacterium* colonization (106). Similarly, in preclinical animal studies, maternal gut microbiome modulation with *Lactobacillus johnsonii* can regulate offspring immunity to RSV, decreasing inflammatory metabolites and protecting neonates against airway immunopathology via metabolic reprogramming (43).

Conversely, early and aberrant colonization by specific bacteria (e.g., *Haemophilus influenzae*, *Streptococcus pneumoniae*, *Moraxella catarrhalis*) in the infant nasopharynx is strongly associated with an increased risk of developing asthma. For instance, preclinical models show that intranasal colonization with nontypeable *H. influenzae* (NTHi) specifically within 4 weeks of birth promotes aggressive immune reprogramming. It drastically amplifies subsequent ovalbumin-induced juvenile airway disease, depleting *FOXP3* gene expression while surging IL-5 and IL-13 production (117). This early pathogenic colonization is heavily influenced by maternal prenatal immunity (e.g., exhibiting low IFN-γ:IL-13 ratios in cord blood) and drives local DNA methylation changes (microbiome-associated differentially methylated regions, mbDMRs) in genes related to immune regulation (e.g., *CDH9*, *PTCHD4*). This creates a pathological, pro-inflammatory microenvironment highly susceptible to chronic airway disease (77, 107, 117–119).

### 3.3 Trained immunity: epigenetic memory in innate immune cells

An emerging and critical concept in asthma and allergy pathogenesis is “trained immunity”—the *de facto* immunological memory exhibited by innate immune cells (macrophages, monocytes, DCs, and ILCs) (102, 120–122). Unlike classical adaptive memory mediated by somatic hypermutation and clonal expansion, trained immunity is fundamentally mediated by profound epigenetic and metabolic reprogramming (e.g., shifts in glycolysis and histone marks like H3K4me3 and H3K27ac) following an initial primary stimulus. This alters the innate cell’s baseline transcription, significantly augmenting its responsiveness to secondary, often completely heterologous, stimuli (65, 122–124). For instance, Hepatitis B Virus (HBV) exposure *in utero* surprisingly triggers a state of trained immunity in newborns, characterized by massive innate immune cell maturation, Th1 development, and elevated IL-12p40 levels, heavily enhancing their ability to respond to heterologous bacterial infections (121).

Early-life systemic viral infections, such as enterovirus (EV-A71) or severe RSV, can detrimentally “train” bone marrow-derived macrophages and DCs. RSV-induced TSLP profoundly alters the chromatin structure in DCs. Specifically, ATAC-seq reveals a gain in physical chromatin accessibility near type 1 IFN genes and differential accessibility at IRF4 and STAT3 binding sites (96). This biases them toward a hyperactive trained state that drives naive T-cells toward pathogenic Th2/Th17 differentiation upon later contact with environmental allergens like house dust mite (HDM) (96, 125). Interestingly, a critical ‘window of opportunity’ in early childhood defines whether this memory function of innate cells drives lifelong disease progression (e.g., transitioning from viral preschool wheeze to chronic asthma) or protective immune regulation (10).

Conversely, exposure to traditional farm environments, raw cow’s milk, or specific bacterial lysates induces a *protective* form of trained immunity (109, 126). The administration of standardized, pharmacological-grade bacterial lysates derived from human airway bacteria represents another frontier. These lysates abrogate allergic lung inflammation by targeting multiple innate immune pathways, including the airway epithelium/IL-33/ILC2 axis and driving the Myd88/Trif-dependent tolerogenic reprogramming of dendritic cells (126). Notably, interferons (IFNs) serve as central orchestrators here; variations in IFN response patterns act as discrete endotypes operating systemically through a lung-blood-bone marrow axis to establish immune training via bacterial lysate immunotherapies (127). Intranasal administration of *Acinetobacter lwoffii* protects against experimental asthma in mice through an IL-6-mediated epigenetic activation of IL-10 production in CD4+ T-cells. In these animal models, this dynamically alters the host’s epigenetic landscape to favor immune tolerance and is accompanied by beneficial, homeostatic shifts in the intestinal microbiome (109).

Beyond microbial lysates, specific adjuvant combinations, such as the synergistic pairing of TLR9 and STING agonists (CpG + 2,3-cGAMP), have proven capable of unlocking robust antigen cross-presentation. This uniquely promotes the expansion of vaccine-specific CD4+ and CD8+ T cells and selectively favors the production of Th1-polarizing cytokines (like IL-12p70 and TNF) over inflammatory IL-6 in neonatal and elderly populations, offering a highly tailored approach to correct early-life immune imbalances (123). Preexposure to CpG oligodeoxynucleotides in early life can likewise mitigate the Th2-skewing effects of neonatal RSV infection, effectively redirecting the immune system towards a protective Th1 response (94). Even maternal immunizations play a role; administration of heterologous Tdap and influenza vaccines during pregnancy has been shown to reprogram infant and maternal peripheral blood mononuclear cells (PBMCs), reducing generalized IL-1β, IL-6, and IL-8 responses to non-specific pattern recognition receptor (PRR) ligand stimulation via trained epigenetic memory (128).

It is also vital to consider age-dependent dynamics. While children and adults mount robust monocyte responses (overexpression of CD169 and PD-L1) to viral immune complexes (ICs), elderly individuals exhibit dysregulated and diminished responses, likely due to immunosenescence and chronic inflammation, highlighting the unique plasticity of the pediatric immune system (101, 129). In murine models, asthmatic lung pathology triggered by Sendai virus is highly age-sensitive, robust in juvenile mice but attenuated in mature mice, driven by an age-dependent evolution of the alveolar macrophage compartment (101).

## 4 Core mechanisms: epigenetic driven airway epithelial barrier and immunometabolism

### 4.1 Epigenetic dysregulation of the airway epithelial barrier

The airway epithelium is not merely a passive physical barrier; it is a dynamic, central immunological sensor that actively orchestrates mucosal immune responses (38, 41, 130–132). Certain children harbor inherent epithelial vulnerabilities, developed *in utero* via epigenetic factors, which result in dysregulated barrier function that perfectly parallels the pathogenesis seen in eosinophilic esophagitis (EoE) (41). In asthmatic children, the epithelium exhibits profound barrier dysfunction, dysregulated repair mechanisms, and chronic, smoldering inflammation (41, 132, 133). This pathology is heavily governed by epigenetic mechanisms. For instance, airway epithelial cells from asthmatic subjects display globally elevated H3K18 acetylation and H3K9 trimethylation at the promoters of key regulatory genes, including *EGFR*, *STAT6*, and *ΔNp63*, which are integral to epithelial differentiation, proliferation, and inflammatory signaling (134). Elevated H3K9me3 levels have also been globally associated with poor growth and immune dysregulation in early infancy (135). In palatine tonsils, differential expression of acetylated histone H3 Lys9 reflects immunological differences between young and aged tissues in chronic inflammation (37). Extending this finding, experimental neonatal colonic inflammation triggers an epigenetic hyper-susceptibility mediated by histone H4K12 hyperacetylation at the *IL1B* promoter, heavily sensitizing the epithelium for massive, life-long immune hyper-reactivity heavily driven by stress hormones (epinephrine) (136).

Emerging evidence also highlights the critical role of Wingless/integrase-1 (Wnt) signaling in this context. Wnt signaling is fundamental for normal embryonic lung development, but recent genetic and epigenetic studies firmly link its aberrant postnatal activation to the pathogenesis and severity of pediatric allergic asthma. Mechanistic models demonstrate that dysregulated Wnt signaling directly drives allergic airway inflammation and structural remodeling, positioning it as a highly promising, novel therapeutic target to halt disease progression (137). More broadly, epigenetic modifications such as DNA methylation and histone acetylation are extensively involved in regulating allergic inflammation by orchestrating the complex interplay between eosinophils, mast cells, and epithelial target cells, which collectively produce inflammatory mediators like reactive oxygen species and chemokines (33). Notably, these mechanistic insights into the respiratory epigenome increasingly rely on *in vitro* models, such as air-liquid interface (ALI) cultures and patient-derived airway organoids, which accurately recapitulate the complex pseudo-stratified epithelial architecture and its epigenetic dynamics.

Genome-wide DNA methylation profiling reveals that genes essential for extracellular matrix (ECM) deposition and wound healing, such as Fibronectin (*FN1*), are aberrantly hypermethylated and repressed, directly contributing to the defective epithelial repair phenotype distinctly observed in asthma (138). In assessing these epigenetic changes, targeted cellular deconvolution confirms that the unique DNA methylation signature of airway epithelial cells (AECs) is fundamentally distinct from PBMCs. Specifically, robust differential methylation of sites within *STAT5A* (decreased expression) and *CRIP1* (elevated expression) perfectly demarcates asthmatic AECs from healthy controls, emphasizing the tissue-specific nature of local epigenetic disease drivers (39).

Exposure to air pollutants (e.g., PM2.5, black carbon, cadmium) profoundly exacerbates this epithelial fragility by altering the methylation status of genes associated with tight junctions (e.g., *CLDNs*) and upregulating pro-inflammatory signaling cascades (*TGFB1*, *IL13*, *CLCA1*, *SYCP2*) (72, 74, 132, 139, 140). Long-term PM2.5 exposure in youth with asthma specifically upregulates *CLCA1* expression in the nasal epithelium, linking directly to biomarkers of T2-high immunity such as blood eosinophils and elevated total IgE (140). Critically, these environmental pollutants, particularly ozone (O3) and endotoxins, synergistically amplify airway inflammation by heavily engaging Toll-like receptor 4 (TLR4) and launching massive inflammasome-mediated signaling cascades driven by uncontrolled oxidative stress (141, 142).

Conversely, protective epigenetic marks can be therapeutically induced; for example, IFN-γ priming enhances the epigenetic upregulation of the crucial viral sensor *DDX58* (RIG-I) by reducing repressive H3K9me3 marks (involving the lysine methyltransferase G9a) at its promoter, thereby significantly boosting the epithelium’s intrinsic antiviral competence against infections like RSV (143). Moreover, epigenetic regulation via non-coding RNAs, particularly microRNAs (e.g., miR-21, miR-146a), acts to modulate these inflammatory pathways, with specific miR-146a mimics successfully reducing eosinophilic inflammation via NF-κB inhibition (132).

### 4.2 Epigenetic and metabolic networks in T-cell polarization (Th1/Th2/Th17/Treg)

Allergic asthma is classically driven by a profound imbalance in Th1/Th2/Th17 lineages and impaired regulatory T (Treg) cell suppressive function (10, 33). The lineage commitment, plasticity, and stability of these cells are strictly governed by epigenetic modifications (25, 33). Hypermethylation of the *RUNX3* promoter silences its expression, tipping the transcription factor balance toward a pathological Th2 shift (increasing IL-4, decreasing IFN-γ) in patients with bronchiolitis progressing to childhood asthma (95). In inner-city asthma cohorts, distinct hypomethylation marks in core TH2 immunity loci, such as *IL13* and the T-lymphocyte regulator *TIGIT*, alongside critical oxidative stress genes, correlate heavily with exorbitant serum IgE concentrations (144). In contrast, DNA methylation of the β-2 adrenergic receptor gene (*ADRB2*) promoter directly associates with significantly decreased asthma severity and reduced dyspnea, showcasing epigenetic modulation of bronchodilator receptors (31). Similarly, increased DNA methylation at the *IL2* promoter (Site 1) at birth strongly predicts childhood asthma exacerbations and asthma-related hospitalizations up to age 8 (124).

Normal Treg suppressive function is critically dependent on the stable demethylation of the *FOXP3* promoter, a process that can be disrupted by environmental pollutants or successfully restored by specific allergen immunotherapies (72, 145). Complicating this picture, severe asthma-associated genetic variants, such as the *IL4R* (encoding IL-4Rα) R576 polymorphism, can exacerbate airway inflammation by recruiting the GRB2 adaptor protein and activating IL-6/STAT3 pathways. This genetic-epigenetic interaction promotes the aberrant and dangerous conversion of induced Tregs (iTregs) into pathogenic Th17-like cells (146). The cAMP response element modulator (*CREM*) is also epigenetically repressed in asthmatics. *CREM* deficiency in T cells results in enhanced Th2 effector cytokines and stronger airway hyperresponsiveness, identifying *CREM* as a critical negative regulator of the Th2 response (147). In murine models, PI3Kδ (e.g., *Pik3cdE1020K* mutation) acts as a central node, where hyperactivation sustains FoxO1 phosphorylation and alters the chromatin landscape to promote massive CD8+ effector T cell differentiation at the expense of central memory cells, providing insight into viral susceptibility in asthma and immunodeficiencies (148).

Intriguingly, epigenetic regulation is tightly coupled with cellular metabolism, a burgeoning field known as immunometabolism (6, 116, 149, 150). Crucially, intracellular metabolites serve as direct, indispensable substrates for epigenetic modifying enzymes. For instance, acetyl-coenzyme A (acetyl-CoA) is the obligate acetyl donor for histone acetyltransferases (HATs), while S-adenosylmethionine (SAM) acts as the universal methyl donor for DNA methyltransferases (DNMTs) and histone methyltransferases. Consequently, the metabolic reprogramming observed during immune cell activation, such as shifts in glycolysis or the tricarboxylic acid (TCA) cycle, physically and directly drives the dynamic remodeling of the epigenetic landscape by altering the local availability of these critical substrates. In the context of early-life undernutrition or extreme metabolic stress, upregulated mTORC1-dependent glycolysis in CD4+ T cells subsequently induces specific DNA hypomethylation in the Th2 cytokine locus, effectively locking the cells into a highly pro-asthmatic Th2 fate (151). In the burgeoning context of obese-asthma, a notoriously difficult-to-treat, non-T2 dominant endotype, metabolic dysregulation triggers profound systemic Th1 polarization, overwhelmingly driven by specific epigenetic upregulation of genes mapping directly to the *CDC42* RhoGTPase pathway (6, 152). Additionally, the massive reprogramming of other metabolic pathways, including tryptophan (specifically upregulating the kynurenine pathway via indoleamine 2,3-dioxygenase 1 and downregulating indole derivatives) and sphingolipid metabolism, is profoundly associated with pediatric asthma severity, immune imbalance, and systemic inflammation during infections like *Mycoplasma pneumoniae* (116, 150, 153).

Notably, the Krebs cycle intermediate, itaconate, acts as a superstar immunometabolite during macrophage inflammatory activation, epigenetically bridging local cell metabolism with powerful anti-inflammatory and electrophilic stress responses (154). Emerging research into Combined Allergic Rhinitis and Asthma Syndrome (CARAS) also reveals the critical roles of N6-methyladenosine (m6A) RNA methylation and ferroptosis in driving the microscopic regulatory cascades that sustain airway hyper-responsiveness (155). Autophagy, another critical homeostatic and metabolic process, has been shown to drive DC over-maturation. Increased LC3-II/I ratios and p62 dynamics in HDM-induced DCs lead to aberrant T-cell differentiation (enhancing Th2/Th17 polarization). Inhibition of autophagy (e.g., via 3-MA) restrains this over-activation and rebalances T-cell responses (149). Likewise, microRNAs dynamically alter gene networks critical for tissue remodeling and inflammation. In early-life pneumovirus infection models, microRNAs drastically downregulate genes such as *GATA3* and *KITLG* (Kit ligand), fundamentally cementing the commitment to a Th2 immunological response (24, 40).

### 4.3 Emerging neuro-immune-epithelial interactions

The nervous system intimately cooperates with mucosal immunity, forming a complex and highly responsive neuro-immune-epithelial axis (156). Recent breakthroughs demonstrate that developing sympathetic nerves in the lung produce dopamine, which signals directly through the DRD4 receptor located on Th2 cells (157). This localized dopaminergic signaling epigenetically facilitates type 2 cytokine expression and is absolutely essential for the robust establishment of allergen-specific Th2 resident memory cells (TRMs) in the immature lung following early-life allergen exposure (157, 158).

Beyond local neuronal signals, profound psychological stress and exposure to violence upregulate neuro-immune coregulation pathways. Such distress (assessed via the Checklist of Children’s Distress Symptoms [CCDS] scale) significantly alters nasal transcriptomic profiles, specifically upregulating potent chemokines like *CCL2*, *CCL8*, *CXCL9*, *CXCL10*, and *S100A7A*, and strongly predisposes affected, minority youth to the highly inflammatory, neutrophilic, T17-high asthma endotype (7, 50, 159). Moreover, specific cellular polarization states are regulated by neuro-endocrine networks. For instance, alveolar macrophage polarization exhibits distinct sex differences during asthma. Estrogen signaling via estrogen receptor alpha (ERα) robustly enhances IL-4-induced M2 macrophage polarization and promotes transcriptionally active histone modifications at M2 gene promoters (81). This estrogen-driven epigenetic regulation directly contributes to the well-documented “sex-switch” in pediatric asthma, where asthma prevalence is significantly higher in pre-pubertal boys but sharply transitions to a higher incidence and severity in post-pubertal females (81).

## 5 Clinical translation: from multi-omics signatures to immune endotypes and precision interventions

### 5.1 Stratifying asthma immune endotypes via multi-omics

Pediatric asthma is no longer treated clinically as a monolithic disease; it consists of highly varied endotypes, primarily divided into T2-high (characterized by profound eosinophilia, high total and specific IgE, and a strong atopic background) and T2-low/T17-high (often characterized by neutrophilia, non-atopic mechanisms, severe exacerbations, or obesity-association) (3–5, 105). State-of-the-art multi-omics approaches, encompassing transcriptomics, epigenomics, and metabolomics, have advanced our ability to accurately stratify these patients and hold promise for predicting disease trajectories (48, 160–164) (**Table 1**).

**Table 1:** Key epigenetic biomarkers associated with pediatric asthma endotypes and exposures.

Biomarker target (gene/locus)	Epigenetic modification	Associated exposure/phenotype	Clinical utility/mechanism	References
RUNX3	Promoter hypermethylation	Bronchiolitis progression to asthma	Skews Th1/Th2 balance; predictive marker for asthma	(95)
IL2 (Site 1)	Promoter hypermethylation	*In utero* environment	Predicts childhood asthma exacerbations and hospital admissions	(124)
FOXP3	Promoter demethylation	Allergen Immunotherapy (SLIT/EPIT)	Stabilizes memory regulatory T cells (Tregs)	(72, 145)
FN1 (Fibronectin)	Repressed expression	Asthma airway remodeling	Dysregulated epithelial wound repair	(138)
VNN1 (Vanin-1)	Promoter differential methylation	Systemic corticosteroids	Discriminates poor vs. good steroid treatment response	(165)
CREB3L1, KSR1	CpG hypomethylation	Albuterol treatment	Reflects epigenomic response to bronchodilators	(166)
TET1	Loss of promoter methylation	Traffic-related air pollution (TRAP)	Dual biomarker for asthma presence and pollution exposure	(167)
CDHR3, CDH26	Differential methylation	Atopy and atopic asthma	Classifies T2-high endotypes via nasal epithelium panels	(4, 163, 168)

For example, comprehensive transcriptomic analysis of the nasal epithelium reveals that T2-high asthma is robustly associated with *IL13* signaling pathways, while T17-high asthma correlates strongly with *IL17* and neutrophil signaling cascades (4). Advanced expression quantitative trait methylation (eQTM) analyses demonstrate that both *cis*- and *trans*-DNA methylation markers extensively regulate airway epithelial gene expression, acting through vast transcription factor and microRNA networks (e.g., targeting *HDC*, *NLRP3*, *ITGAE*) (163, 169). In specific eQTM investigations, highly complex interactions emerge; for example, locus-locus interaction analyses show that DNAm levels of *CDH6* and *RAPGEF3* actively cross-talk to jointly predict absolute asthma risk, establishing central AECs as demonstrably superior to PBMCs for robust diagnostic accuracy (170). At a genome-wide scale, rigorous functional genomics pipelines utilizing whole-genome bisulfite sequencing have mapped out bespoke “high-value” Asthma&Allergy CpGs that capture functional promoter loops, proving profoundly robust across extreme geographical and racial disparities (171).

Importantly, large-scale integrative genomic studies have identified that genetic variations (e.g., SNPs at loci like *ADAMTS1*, *RAD54B*, *EGLN3*) strongly influence these epigenetic-defined endotypes. Specifically, within diverse populations such as African American youth, novel loci linked strictly to admixture mapping and fine epigenetic fine-mapping distinctly outline the genetic boundaries of post-bronchodilator lung function phenotypes (172). The joint SNP heritability of these epigenetic signatures is highly significant (e.g., 0.26 for atopy signatures), indicating that a substantial portion of endotypic susceptibility is hardwired at birth, dictating how the child will epigenetically respond to the early-life environment (26, 27, 172).

### 5.2 The nasal epigenome as a biomarker for distal airway inflammation

The nasal epithelium serves as an easily accessible, non-invasive surrogate proxy for the lower respiratory tract (the bronchial epithelium), making it a highly promising source for discovering potential clinical biomarkers in pediatric populations (171, 173–175). EWAS utilizing simple nasal brushings have identified promising DNA methylation panels (often involving merely three to a few dozen CpGs) that show potential to classify atopy, aid in diagnosing asthma, detect allergic multimorbidity, and correlate highly with FeNO levels (168, 176–180). Specific hypomethylation of I*L6* and *NOS2* (iNOS) in nasal cells directly correlates with skyrocketing FeNO levels (180). Further, nasal EWAS panels flag profound differential methylation in genes strictly implicated in eosinophilia like *EPX*, *IL4*, and *IL13*, effectively serving as potential molecular indicators of local airway inflammation (175). However, it is important to note that despite these promising discoveries, most proposed nasal epigenetic biomarkers currently remain research tools. Their routine clinical implementation is pending, as broad reproducibility across diverse populations and large-scale prospective validation studies are still required. Deep profiling of severe African American childhood asthma has identified over 816 differentially methylated positions (DMPs) in the nasal epithelium, powerfully segregating severe from non-severe disease phenotypes (181). Blood-based multi-omics networks similarly yield tremendous diagnostic yield. For example, dysregulation of *TNF*, *HLA*-*DPA1*, and miR-148b creates a potent biomarker triad predicting atopic asthma immune responsiveness (164), while co-expression networks centered around *CCL2* and miR-206 precisely map the cytokine-receptor interactions underlying childhood asthma severity (162). MicroRNAs also serve as powerful biomarkers; for instance, reduced circulatory miR-296-5p and miR-16-5p levels are strongly associated with airway hyperresponsiveness (methacholine PC20) and regulate airway smooth muscle phenotypes (182). To further enhance resolution, computational deconvolution of bronchoalveolar lavage (BAL) fluid has been meticulously modeled using defined DNA methylation profiles to separate out specific signals from alveolar macrophages, granulocytes, and lymphocytes, enabling hyper-accurate mixed-cell pulmonary studies (183). In infant bronchiolitis, systemic epigenetic analyses flag severe *IL1RL1* (ST2 protein) pathway hyper-activation as directly causal to asthma progression (184, 185).

Beyond diagnosis, emerging evidence suggests specific DNA methylation marks may correlate with therapeutic responses, highlighting early steps toward personalized pediatric pharmacotherapy (165). For instance, differential methylation in the *VNN1* (vanin-1) promoter reliably discriminates poor versus good responders to systemic corticosteroids in acute pediatric asthma exacerbations (165). Correspondingly, acute steroid treatment dynamically modifies nasal DNAm; explicitly, hypomethylation in the *OTX2* and *LDHC* promoters perfectly differentiates “good responders” discharged from the Emergency Department under 24 hours (179). Similarly, specific CpG sites mapped to genes like *CREB3L1*, *MYLK4*, and *KSR1* reflect dynamic epigenomic responses to albuterol (a common short-acting beta-agonist bronchodilator) (166). Peripheral blood DNA methylation profiles (e.g., relative hypermethylation of cg27254601 linked to *BOLA2*) also correlate significantly with the long-term clinical efficacy and lung function improvement (FEV1 change) observed with inhaled corticosteroids (ICS) (182, 186). Additionally, loss of methylation at the *TET1* promoter serves as a highly specific dual biomarker for both asthma presence and the magnitude of exposure to TRAP (167). To study these markers directly *ex vivo*, scientists have successfully generated induced pluripotent stem cells (iPSCs) derived from pediatric nasal epithelial cells. These cells retain epigenetic memory of their asthmatic tissue of origin, creating valuable models for personalized drug screening (187).

### 5.3 Future therapeutic strategies targeting epigenetics and microbiota

A deep, mechanistic understanding of the epigenetic-immune-microbiome axis opens unprecedented, novel avenues for asthma prevention and precision therapy (22, 188).

#### 5.3.1 Epigenetic modulators

Preclinical *in vitro* and *in vivo* models show that inhibiting histone deacetylases (HDACs) or DNA methyltransferases (DNMTs, using hypomethylating agents like 5-azacytidine or decitabine) can partially restore epithelial barrier function, modulate cytokine secretion (decreasing IFN-γ while significantly elevating IL-10), and alleviate aberrant Th2 responses in CD4+ T cells, reversing the hyporesponsive neonatal immune state (100, 132, 136, 189). The specific EZH2 inhibitor GSK126 has been shown to effectively attenuate post-inflammatory wheezing by blocking NF-κB-related H3K27me3 hypermethylation in alveolar macrophages (190). At the epitranscriptomic level, editing A-to-I RNA transcripts via ADAR enzymes (targeting genes like *CCRL2* and *CCNI*) offers a novel epitranscriptomic route to modulate innate immunity in complicated pneumonia (119).

#### 5.3.2 Microbial and dietary interventions

Modulating the gut-lung axis via specific probiotics (e.g., *Lactobacillus johnsonii*), prebiotics, or specific dietary regimens (such as the Mediterranean or Ketogenic diets) shows immense promise in restoring immune balance and mitigating the Th2/Th17 skew (43–45). Striking comparative studies between Russian Karelia and Finnish Karelia, regions with starkly contrasting environments, prove that rich microbial biodiversity fosters a profoundly tightly integrated immune-metabolic network. High circulating SCFAs directly suppress inflammatory epigenetic RNA modules, naturally shielding against atopy (116). Clinically, maternal vitamin C supplementation during pregnancy in heavy smokers directly alters offspring buccal DNA methylation at critical loci (e.g., *SLC25A37*), significantly preserving lung function (FEF25-75) measured at 5 years of age (11, 49). Epigenetic profiling in infection- and asthma-prone infants (IAP) identifies a low vaccine responsiveness (LVR) phenotype that can be partially reversed by stimulation with TLR agonists like monophosphoryl lipid A (MPL), highlighting the potential for immunomodulation via adjuvants (191).

#### 5.3.3 Allergen immunotherapy

Both sublingual immunotherapy (SLIT) and epicutaneous immunotherapy (EPIT) exert long-lasting clinical tolerance. This profound tolerance is achieved not merely by transiently shifting cytokine profiles, but by inducing deep, durable epigenetic modifications. These include the significantly increased DNA methylation of the *IL4* promoter and the sustained demethylation of the *FOXP3* locus, which securely stabilizes the suppressive function of memory Tregs against the specific allergen (e.g., *Dermatophagoides pteronyssinus* specific tolerance) (145, 192, 193). Furthermore, preclinical evidence suggests EPIT may alter the progression of the allergic march; treating milk-sensitized murine models with EPIT has been shown to prevent subsequent secondary sensitization to peanut or house dust mite via Treg-dependent hypermethylation of the *GATA3* promoter region (193).

### 5.4 Knowledge gaps and methodological challenges

#### 5.4.1 Methodological challenges in epigenetic causal inference

While multi-omics approaches offer profound insights into pediatric asthma, establishing true causality from epigenetic data remains methodologically challenging (194). First, many reported associations between epigenetic signatures and asthma risk may be influenced by reverse causation (e.g., the disease state or pharmacotherapy altering the epigenome rather than the reverse), residual confounding, and unmeasured environmental or genetic factors (195). Second, EWAS are particularly susceptible to bias arising from differences in cell-type composition between cases and controls (196, 197). Because epigenetic marks are highly cell-type specific, shifts in local immune cell populations can generate apparent epigenetic signals that merely reflect cellular heterogeneity rather than underlying disease mechanisms (195, 196).

Compounding these issues, while mediation analyses are frequently employed to link early-life exposures to asthma via epigenetic modifications, interpreting these models requires caution, as they often rely on strong statistical assumptions that are not fully testable in purely observational cohorts (198, 199). Additionally, the tissue-specific nature of epigenetic marks raises critical questions regarding the extent to which findings from easily accessible surrogate tissues, such as peripheral blood or the nasal epithelium, accurately reflect the precise disease-relevant processes occurring in the lower airways (200). Addressing these methodological limitations is essential to provide a balanced assessment of current evidence, helping to accurately distinguish between purely correlative epigenetic biomarkers, downstream consequences of the disease, and true causal mechanisms driving asthma pathogenesis (194).

#### 5.4.2 Translational barriers and research priorities

Despite the successful identification of numerous promising epigenetic and transcriptomic biomarkers, their rapid translation into routine clinical diagnostics is heavily hindered. The primary obstacle is the lack of standardized, cost-effective, high-sensitivity analytical platforms validated across diverse, multi-ethnic, real-world pediatric cohorts (155, 170, 181, 191). Currently, much of the available evidence has been generated in relatively limited and population-specific cohorts. Therefore, future research must give much greater attention to the ethnic and geographic generalizability of these epigenetic findings (27, 171, 172). A major hurdle remains the critical need for absolute standardization and reproducibility. Current epigenetic biomarkers are frequently derived from disparate analytical platforms (e.g., Illumina 450K versus EPIC arrays) and rely on heterogeneous bulk tissue mixtures (such as whole blood or crude nasal swabs). To overcome the background noise generated by this cellular heterogeneity, future diagnostic pipelines must pivot towards single-cell DNA methylation sequencing (scDNAm-seq) and spatial transcriptomics. Equally critical, robust longitudinal interventional trials are urgently required to definitively confirm whether modifying the microbiome (e.g., via fecal microbiota transplantation or specific bacterial lysates) can durably “erase” established pathogenic epigenetic marks in human asthma. Specifically, tracking lung function trajectories through adolescence, where distinct, gender-specific methylation changes mapped to key genes are heavily active, demands continuous, unbroken surveillance spanning from birth through puberty to realize true interventional success (201).

Looking ahead, several critical unresolved questions must direct future investigations. It is imperative to distinguish whether identified epigenetic changes are true causal drivers of asthma pathogenesis or merely downstream biomarkers of chronic inflammation. The long-term temporal stability of these epigenetic signatures across the lifespan also requires rigorous evaluation. Crucially, it remains unknown to what extent these pathogenic epigenetic programs can be safely and durably reversed in humans. Finally, the true clinical utility of these proposed multi-omics biomarkers must be critically assessed to determine if they provide actionable advantages over existing, conventional asthma risk prediction tools.

## 6 Concluding remarks and future perspectives

The inception of pediatric asthma is a deeply complex, multifactorial process orchestrated by the dynamic interplay between environmental exposures, the maturing microbiome, and host immunity during the critical first 1,000 days of life. As comprehensively synthesized in this review, *in utero* exposures, such as maternal psychological stress, high-fat diets, pollution, and maternal immune activation, lay the foundational epigenetic groundwork, establishing an aberrant baseline of immune susceptibility. Postnatal “second hits”, primarily in the form of severe viral lower respiratory tract infections (RSV, HRV) and microbial dysbiosis, readily exploit this pre-existing vulnerability. These combined hits trigger epigenetic and metabolic reprogramming within the airway epithelium and innate immune cells (a process termed trained immunity), which may contribute to driving distinct T-cell polarization and epithelial barrier dysfunction that characterize clinical asthma endotypes (T2-high vs. T17/T2-low).

Nevertheless, while the epigenetic-immune-microbiome axis presents a compelling emerging framework, much of the current supporting evidence remains associative, mechanistic, or derived from preclinical models. Translating these insights into clinical applications requires caution and underscores the need for rigorous mechanistic validation, extended longitudinal investigation, and robust interventional confirmation in human populations.

Building on these considerations, future research must shift from cross-sectional observational studies to longitudinal, systems-biology approaches. Leveraging large, well-phenotyped birth cohorts (e.g., AERIAL, ORIGINS, and INSPIRE) (26, 60, 131), integrated with cutting-edge single-cell sequencing, spatial transcriptomics, and multi-omics networks (such as mbEWAS), will be essential (107). To integrate these massive, high-dimensional multi-omics datasets, future analytical pipelines will need to rely on advanced artificial intelligence (AI) and machine learning (ML) algorithms. By constructing complex biomarker networks through these computational approaches (as illustrated in **Graphical Abstract**), clinicians may eventually be better equipped to stratify patients into immune endotypes and predict individual therapeutic responsiveness and long-term asthma trajectories. Only by decoding the intricate epigenetic-immune-microbiome axis at single-cell resolution can we develop novel, mechanism-based therapeutics. Targeted epigenetic modulators, immunometabolic reprogrammers, and microbiome-derived adjuvants hold the potential not only to manage clinical symptoms but to target the developmental origins and potentially modify the trajectory of pediatric asthma.

## Glossary

AECs: Airway epithelial cells
AI: Artificial intelligence
AIT: Allergen immunotherapy
APPs: Antimicrobial proteins
BAL: Bronchoalveolar lavage
BBP: Butyl benzyl phthalate
CCDS: Checklist of Children’s Distress Symptoms
Cd: Cadmium
cfDNA: Cell-free DNA
CHILD: Canadian Healthy Infant Longitudinal Development
COAST: Childhood Origins of ASThma
COPSAC: Copenhagen Prospective Studies on Asthma in Childhood
CpG: Cytosine-phosphate-guanine
DC: Dendritic cell
DEHP: Di-(2-ethylhexyl) phthalate
DNAm: DNA methylation
DNMTs: DNA methyltransferases
DOHaD: Developmental origins of health and disease
EDCs: Endocrine-disrupting chemicals
EMT: Epithelial-to-mesenchymal transition
EPIT: Epicutaneous immunotherapy
eQTM: Expression quantitative trait methylation
ERα: Estrogen receptor alpha
EWAS: Epigenome-wide association study
FeNO: Fractional exhaled nitric oxide
FEV1: Forced expiratory volume in 1 second
FVC: Forced vital capacity
HATs: Histone acetyltransferases
HDACs: Histone deacetylases
HDM: House dust mite
HMPV: Human metapneumovirus
HPA: Hypothalamic-pituitary-adrenal
HRV: Human rhinovirus
ICS: Inhaled corticosteroids
IFN: Interferon
IgE: Immunoglobulin E
ILC2s: Group 2 innate lymphoid cells
LRTI: Lower respiratory tract infections
mbDMRs: Microbiome-associated differentially methylated regions
MIA: Maternal immune activation
MIS-C: Multisystem Inflammatory Syndrome in Children
ML: Machine learning
mTORC1: Mechanistic target of rapamycin complex-1
ncRNAs: Non-coding RNAs
NTHi: Nontypeable *Haemophilus influenzae*
PASTURE: Protection against Allergy, Study in Rural Environments
PBMCs: Peripheral blood mononuclear cells
PM2.5: Particulate matter <2.5 μm
PRR: Pattern recognition receptor
RSV: Respiratory syncytial virus
SAM: S-adenosylmethionine
SCFAs: Short-chain fatty acids
scDNAm-seq: Single-cell DNA methylation sequencing
SLIT: Sublingual immunotherapy
SNPs: Single nucleotide polymorphisms
TCRS: Tucson Children’s Respiratory Study
Th: T helper
TRAP: Traffic-related air pollution
Tregs: Regulatory T cells
TRMs: Tissue-resident memory T cells
URECA: Urban Environment and Childhood Asthma
VDAART: Vitamin D Antenatal Asthma ReductionTrial.
